# Supplementary material for: In vivo quantification of anterior and posterior chamber volumes in mice: implications for aqueous humor dynamics
Source: bioRxiv. 2024 Jul 25:2024.07.24.604989. Preprint. [Version 1] doi: 10.1101/2024.07.24.604989 (PMC11291131; doi:10.1101/2024.07.24.604989)
Supplement: Supplement 1 [file NIHPP2024.07.24.604989v1-supplement-1.pdf]

bioRxiv preprint doi: https://doi.org/10.1101/2024.07.24.604989; this version posted July 25, 2024. The copyright holder for this preprint (which was not certified by peer review) is the author/funder, who has granted bioRxiv a license to display the preprint in perpetuity. It is made available under aCC-BY 4.0 International license.

Supplemental Text

Vis-OCT imaging system: Light from a supercontinuum laser (SuperK EXTREME, NKT Photonics) was filtered using a dichroic mirror (DMSP650, Thorlabs), spectral shaping filter (34-443, Edmund Optics), and bandpass filter (FF02-694/SP-25, Semrock) before being sent to a 90:10 fiber coupler (TW560R2A2, Thorlabs). The reference arm consisted of polarization controllers (FPC560, Thorlabs) and BK7 dispersion compensation glass (27-852, Edmund Optics). Light in the sample arm was scanned using a pair of galvanometer mirrors (Compact-506, ScannerMax) through a 25 mm achromatic doublet scan lens (AC127-025-A, Thorlabs). The light from the scan lens was focused on the sample. Reflected light from the sample arm and the light transmitted through the reference arm was coupled to a second 50:50 fiber coupler (TW560R2F2, Thorlabs). Two spectrometers (Blizzard SR, Opticent Health) operating from 510 nm to 610 nm detected the interferogram signals propagating through the second fiber coupler for image reconstruction. We used two spectrometers for balanced detection to eliminate the influences of relative intensity noise [54]. The axial resolution of the system is 1.3 µm [24], and the lateral resolution is 8.8 µm as measured with a USAF51 target card (R1DS1P, Thorlabs). The vis-OCT's A-line rate was 75 kHz, and the illumination power on the sample was 0.8 mW.

Fusion of individual volumes into a composite volume: A total of eight transformations were obtained from the eight vis-OCT sub-volumes. Using these transformations, we mapped the coordinate system of all volumes to the coordinate system of the first acquired vis-OCT volume (Fig. 2d). For vis-OCT volumes not adjacent to the first volume, the transformation matrices for each volume between the given volume and the first volume can be multiplied to determine the net transformation of the given volume to the first volume. Specifically, given the transformation T_i mapping volume i onto the reference coordinate system, coordinate (x_i, y_i, z_i) in the reference frame of the volume is mapped to (x', y', z') in the reference coordinate system by

$$T_i(x_i, y_i, z_i) = \begin{bmatrix} r_{11} & r_{12} & r_{13} & t_x \\ r_{21} & r_{22} & r_{23} & t_y \\ r_{31} & r_{32} & r_{33} & t_z \\ 0 & 0 & 0 & 1 \end{bmatrix} \begin{bmatrix} x_i \\ y_i \\ z_i \\ 1 \end{bmatrix} = \begin{bmatrix} x' \\ y' \\ z' \\ 1 \end{bmatrix}. \quad \text{Eq. 1}$$

After mapping all eight volumes onto a common reference frame, we identified the overlapping regions between each adjacent volume pair. Next, we applied an iterative closest point (ICP) algorithm to refine the transformation of each volume to the common reference frame[55]. Specifically, we used ICP to minimize the distance between the point clouds of

bioRxiv preprint doi: https://doi.org/10.1101/2024.07.24.604989; this version posted July 25, 2024. The copyright holder for this preprint (which was not certified by peer review) is the author/funder, who has granted bioRxiv a license to display the preprint in perpetuity. It is made available under aCC-BY 4.0 International license.

overlapping regions of adjacent volumes. The purpose of the refinement step was to increase the number of points used to register adjacent volumes and to address the loop closure problem [31], which results from the error propagating from the multiplication of multiple transformation matrices. After refining the transformation, we defined the intensity of the reconstructed signal in the global reference frame V(x',y',z') as

$$T_i(x_i, y_i, z_i) = \begin{bmatrix} x' \\ y' \\ z' \\ 1 \end{bmatrix} \Rightarrow V(x', y', z') = I(x_i, y_i, z_i)\,. \qquad \text{Eq. 2}$$

In other words, if T_i maps (x_i,y_i,z_i) in the original reference frame of volume *i* to (x',y',z'), then the intensity of the reconstructed signal V(x',y',z') is that of I(x_i,y_i,z_i) in the original reference frame of volume *i*. For situations in where the mapped pixel was shared between two adjacent scans, i.e. in which T_i maps (x_i,y_i,z_i) to (x',y',z') and T_j maps (x_j,y_j,z_j) to the same (x',y',z'), we assigned V(x',y',z') as max{I(x_i,y_i,z_i) in the reference frame of volume I, I(x_j,y_j,z_j) in the reference frame of volume j}.

Determination of the approximate anterior hyaloid membrane location: First, we identified the inner boundaries of the anterior and posterior chambers (Fig. 4a) which coincided with the anterior boundary of the lens (Fig. 4b). Next, we fit an ellipsoid to the lens boundary by minimizing the least-squared error (LSE)

$$LSE = \sum_{i=1}^{n}(Ax_i^2 + By_i^2 + Cz_i^2 + Dx_iy_i + Ex_iz_i + Fy_iz_i + Gx_i + Hy_i + Iz_i + J)^2\,, \qquad \text{Eq. 3}$$

where (x_i, y_i, z_i) are the lens boundary points and A-J are coefficients for the general quadric surface equation (Fig. 4c). After fitting, we found the center of the lens ellipsoid (x, y, z) by solving the following equation.

$$-\begin{bmatrix} A & D & E \\ D & B & F \\ E & F & C \end{bmatrix}\begin{bmatrix} x \\ y \\ z \end{bmatrix} = \begin{bmatrix} G \\ H \\ I \end{bmatrix}. \qquad \text{Eq. 4}$$

We determined the optical axis of the eye by using principal component analysis [56] of the coordinates for all segmented voxels corresponding to the anterior chamber. The calculated principal components are orthogonal vectors, with the vector aligning most closely to the z-axis of the reconstructed volume being the direction of the optical axis of the eye. We approximated the anterior hyaloid membrane as the plane passing through the center of the ellipsoid with a normal vector the same as the optical axis of the eye (Fig. 4d).

bioRxiv preprint doi: https://doi.org/10.1101/2024.07.24.604989; this version posted July 25, 2024. The copyright holder for this preprint (which was not certified by peer review) is the author/funder, who has granted bioRxiv a license to display the preprint in perpetuity. It is made available under aCC-BY 4.0 International license.

Finally, we updated the boundaries of the lens and outer surface of the posterior chamber (Fig. 4e) and applied a k-means-based volumetric segmentation on the full volumetric reconstruction [57]. We updated the posterior chamber segmentation using the newly segmented posterior chamber outer boundary, the lens boundary, and the plane approximating the anterior hyaloid membrane, as highlighted by the blue regions in Fig. 4f.

bioRxiv preprint doi: https://doi.org/10.1101/2024.07.24.604989; this version posted July 25, 2024. The copyright holder for this preprint (which was not certified by peer review) is the author/funder, who has granted bioRxiv a license to display the preprint in perpetuity. It is made available under aCC-BY 4.0 International license.

Supplemental Figures

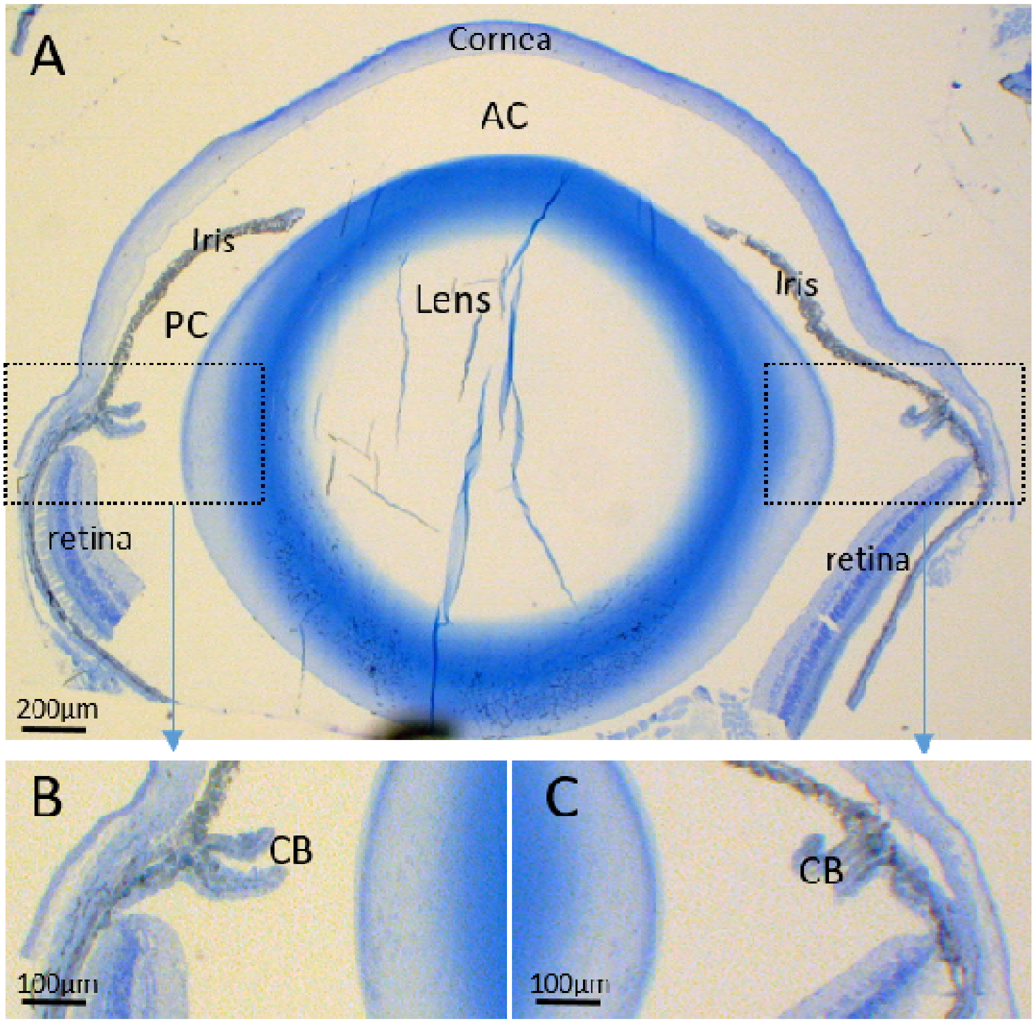

Supplemental Figure S1: A) Histologic cross-section of a mouse eye from a 3-month-old female C57BL/6J wild-type mouse. Although distortion of the globe is evident due to histologic preparation, it is evident that the lens equator coincides approximately with the posterior margin of the ciliary body and the anterior termination of the retina. **B)** and **C)** show zoomed-in views of limbal area showing the left and right sides of the CB and anterior edges of retina. AC: anterior chamber, PC: posterior chamber, CB: ciliary body.

bioRxiv preprint doi: https://doi.org/10.1101/2024.07.24.604989; this version posted July 25, 2024. The copyright holder for this preprint (which was not certified by peer review) is the author/funder, who has granted bioRxiv a license to display the preprint in perpetuity. It is made available under aCC-BY 4.0 International license.

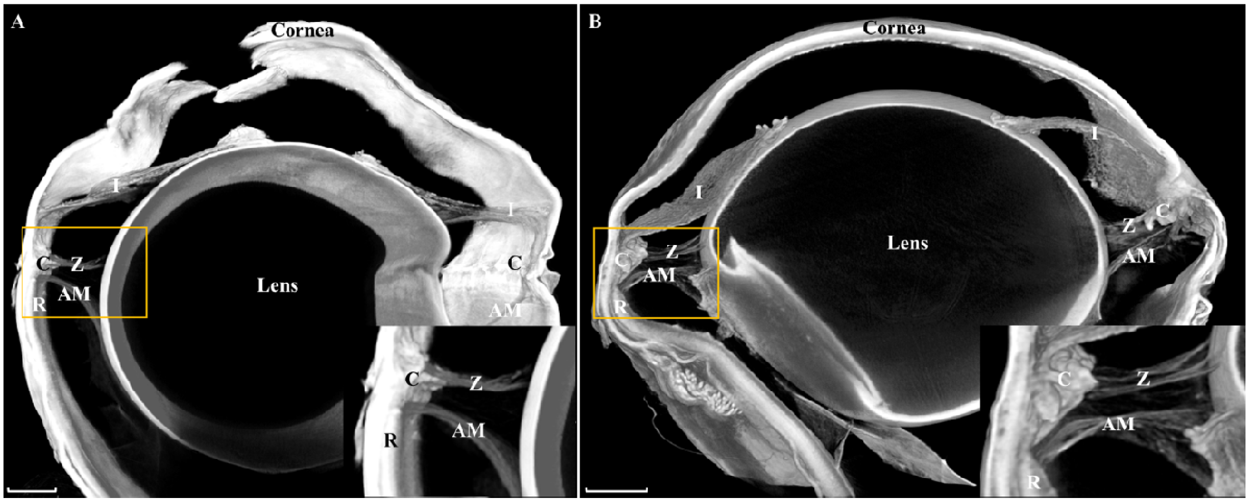

Supplemental Figure S2: Digital reconstructions from 3-dimensonal micro-CT images of an eye from a 13-month-old DBA/2J female mouse **(A)** and a 1.5-month-old C57BL/6J female mouse **(B)**. Scan resolutions are 0.87 µm and 0.75 µm, respectively. In (A), a corneal window was created to facilitate contrast agent penetration. The far side of the eye has been digitally removed to better visualize the structures of interest. Zonules are seen stretching from the ciliary processes to the lens. The peripheral edge of the anterior hyaloid membrane extends to the anterior boundary of the retina. The yellow boxes outline the areas of the insets. AM = Anterior Hyaloid Membrane, C = Ciliary Body, I = Iris, R = Retina, Z = Zonules, Scale bars = 250 µm.
